# Implications of Fumarate Hydratase Deficiency (FHD) and Cancer Risk: A Window into the Clinical and Oncological Implications of a Rare Disorder in Gynecology

**DOI:** 10.3390/cancers17040573

**Published:** 2025-02-08

**Authors:** Marco D’Indinosante, Sara Lardino, Matteo Bruno, Guglielmo Stabile, Matteo Pavone, Gaia Giannone, Pasquale Lombardi, Gennaro Daniele, Francesco Fanfani, Francesca Ciccarone, Giovanni Scambia

**Affiliations:** 1 Dipartimento per le Scienze Della Salute Della Donna, del Bambino e di Sanità Pubblica, UOC Ginecologia Oncologica, Fondazione Policlinico Universitario Agostino Gemelli IRCCS, 00168 Rome, Italy; marco.dindinosante@guest.policlinicogemelli.it (M.D.); s.lardino@ic.ac.uk (S.L.); giovanni.scambia@policlinicogemelli.it (G.S.); 2 Ovarian Cancer Action Research Centre, Department of Surgery and Cancer, Imperial College London, London SW7 2AZ, UK; 3 Department of Medical and Surgical Sciences, Institute of Obstetrics and Gynecology, University of Foggia, 71122 Foggia, Italy; 4 ICube, Laboratory of Engineering, Computer Science and Imaging, Department of Robotics, Imaging, Teledetection and Healthcare Technologies, University of Strasbourg, CNRS, 67081 Strasbourg, France; 5 Division of Cancer, Department of Surgery and Cancer, Imperial College London, Hammersmith Hospital, London SW7 2AZ, UK; 6 Phase 1 Unit, Fondazione Policlinico Universitario A. Gemelli IRCCS, 00168 Rome, Italy

**Keywords:** fumarate hydratase deficiency, HLRCC, leiomyosarcoma

## Abstract

Fumarate hydratase (FH) deficiency is a rare, yet impactful metabolic disorder caused by mutations in the FH gene, affecting the Krebs cycle, leading to the accumulation of fumarate and pseudohypoxic states. This metabolic shift promotes cell signaling alterations that can drive tumorigenesis, as heterozygous germline mutations in the FH gene, resulting in hereditary leiomyomatosis and renal cell carcinoma (HLRCC) syndrome. FH-deficient uterine leiomyomas show peculiar histological features that may lead to misdiagnosis STUMP (smooth muscle tumor of uncertain malignant potential) and uLMS (uterine leiomyosarcoma). Definitive diagnosis involves clinical evaluation, imaging, and histopathological examination, with immunohistochemistry for FH protein being a key diagnostic tool. Management of FH-deficient leiomyomas may involve conventional treatments like surgery and hormonal therapy but also requires careful monitoring and genetic counseling for associated malignancies. High-intensity focused ultrasound (HIFU) has emerged as a promising treatment option for fibroids, although long-term efficacy remains a concern also because of its inability to obtain tissue for a pathological diagnosis. Fumarate hydratase deficiency (FHD) represents a significant challenge in gynecologic oncology due to its association with an increased risk of hereditary leiomyomatosis and renal cell carcinoma. Nevertheless, to the best of our knowledge, there is a lack of studies demonstrating the potential role of FH deficiency in increased risk of leiomyosarcomatosus transformation. Early detection, genetic screening, and personalized treatment approaches are critical for improving patient outcomes. The aim of this review is to develop a narrative overview of the implications of FHD in gynecological diseases and its correlation with cancer risk. For the first time, this review offers an overview of the necessity for studies to address the possible correlation between FH deficiency and the risk of developing leiomyosarcoma, focusing on new perspectives that can be explored in the field of better FH deficiency knowledge and cancer risk.

## Simple Summary

Fumarate hydratase deficiency represents a complex challenge in gynecologic oncology due to its association with an increased risk of both hereditary leiomyomatosis and renal cell carcinoma (HLRCC) and potential (not fully understood) malignant transformation in uterine leiomyosarcomas. Early detection, genetic screening, and personalized treatment approaches are crucial for improving patient outcomes. As our understanding of FH deficiency and its role in tumorigenesis evolves, future research is needed to better define the long-term oncological risks associated with FH-deficient tumors, particularly regarding their potential to undergo transformation into leiomyosarcoma. This review aims to highlight the need for increased awareness and more comprehensive studies on the implications of FH deficiency in gynecological diseases and its broader correlation with cancer risk, which could help guide clinicians in managing these rare but significant conditions.

## 1. Introduction

Fumarate hydratase deficiency (FHD) is a metabolic disorder that affects the Krebs cycle and results in the accumulation of fumarate in the body, disrupting cellular metabolism. This deficiency is caused by mutations in the fumarate hydratase (FH) gene, which could occur in a sporadic and germline setting [1]. Biallelic germline mutation results in fumaric aciduria, a severe disorder characterized by neurological signs and development abnormalities. Heterozygous germline mutation leads to hereditary leiomyomatosis and renal cell carcinoma (HLRCC), a rare autosomal dominant disorder, characterized by the development of multiple cutaneous leiomyomata and RCC, and in females, uterine leiomyomatosis with early onset [1,2,3]. Indeed, FH deficiency in uterine leiomyomas, or fibroids, presents clinically as multiple, large, and symptomatic benign tumors that may differ significantly from sporadic leiomyomas [4]. From the histopathological point of view, these FH-deficient leiomyomas exhibit increased cellularity, nuclear atypia, and a higher mitotic index, contributing to their difficult management, considering their histological analogies with STUMP (smooth muscle tumor of uncertain malignant potential) and uLMS (uterine leiomyosarcoma) [2,4,5]. Genetic screening and morphological evaluation are essential for the identification and management of FH-deficient leiomyomas, as these entities are rare. Increased intracellular fumarate levels resulting from FH deficiency lead to the stabilization of hypoxia-inducible factors (HIFs), promoting a pseudo-hypoxic drive that can enhance tumorigenesis. Hypoxia-inducible factors mostly involved are the constitutive ones, such as HIF-1α and HIF-2α. Furthermore, FH-deficient tumors have been shown to display distinct molecular signatures and immunohistochemical profiles, which help to differentiate them from other leiomyomas [3,6]. The association between FHD and increased renal cell carcinoma risk underpins the need for vigilant monitoring for both cancer and leiomyomatosis, enabling early intervention in healthy carriers. In this setting, patients with FHD and leiomyomas represent a significant clinical challenge due to aggressive tumor characteristics and increased malignancy risk, demanding a personalized and comprehensive diagnostic and therapeutic approach. Understanding the genetic and molecular features of leiomyomas in patients with FHD would facilitate the development of targeted therapies and personalized management strategies, which are critical in improving patient outcomes [3]. Therefore, the aim of this review is to provide an overview of the implications of FH deficiency in gynecological diseases and its correlation with cancer risk. 

## 2. FH Deficiency

**Figure 1:**
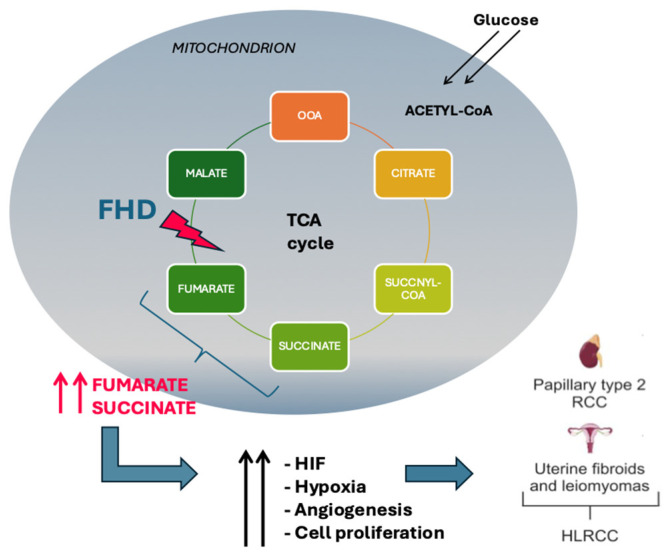
Outcomes of the fumarate hydratase mutation in Krebs cycle. FH activity loss leads to intracellular accumulation of fumarate, causing stabilization of HIF which promotes cell proliferation. HIF: hypoxia-inducible factors; FHD: fumarate hydratase deficiency; HLRCC: hereditary leiomyomatosis and renal cell carcinoma.

Fumarate hydratase (FH) deficiency is a rare metabolic disorder that disrupts the normal function of the citric acid cycle, an essential pathway for cellular energy production. The FH enzyme catalyzes the hydration of fumarate to malate, a critical step in the citric acid cycle. Mutations in the FH gene lead to a loss of enzyme activity, resulting in the accumulation of fumarate within cells. This accumulation causes a cascade of metabolic disorders, impacting cellular respiration and energy production. The citric acid cycle, also known as the Krebs cycle, plays a central role in cellular metabolism by oxidizing acetyl-CoA to carbon dioxide and capturing high-energy electrons in the form of nicotinamide adenine dinucleotide (NADH) and flavin adenine dinucleotide (FAD)H2, which are used in the electron transport chain to generate adenosine triphosphate (ATP). FH deficiency disrupts this cycle, leading to reduced production of ATP and an increased reliance on glycolysis for energy, which is less efficient and produces lactic acid as a byproduct. This shift in metabolism is known as the Warburg effect and is commonly observed in cancer cells [3]. Mutations in the FH gene have been identified in various types of tumors, including hereditary leiomyomatosis and renal cell carcinoma (HLRCC), a condition characterized by the development of multiple benign and malignant tumors. The accumulation of fumarate in cells with FH mutations can inhibit prolyl hydroxylases, leading to the stabilization of hypoxia-inducible factor (HIF), a transcription factor that promotes angiogenesis, cell proliferation, and survival under low oxygen conditions. This aberrant activation of HIF pathways could contribute to tumor growth and survival, providing a crucial link between FH deficiency and oncogenesis. In addition to its role in the citric acid cycle, fumarase has been shown to have tumor suppressor functions, and its loss of function can drive tumorigenesis through various mechanisms, including epigenetic modifications. Accumulated fumarate can act as an oncometabolite, leading to the inhibition of histone and DNA demethylases, resulting in widespread changes in gene expression that favor tumor development and progression [3,6] (Figure 1).

The diagnosis of FH deficiency involves biochemical assays to measure fumarate levels in body fluids and genetic testing to identify mutations in the FH gene. Clinically, patients with germline biallelic loss of FH expression may present a range of symptoms, including developmental delay, hypotonia, and seizures in severe cases, while in the context of HLRCC (heterozygous germline mutation), patients may present with cutaneous and uterine leiomyomas, as well as renal cell carcinoma. Management of FH deficiency-related conditions involves a multidisciplinary approach, including metabolic interventions to manage symptoms and targeted therapies aimed at inhibiting HIF pathways. Research is ongoing to develop novel therapeutic strategies that can address the metabolic vulnerabilities of FH-deficient tumors [7,8]. Understanding the molecular and genetic basis of FH deficiency is crucial for the development of effective diagnostic and therapeutic approaches.

### Somatic and Germinal Prevalence

**Figure 2:**
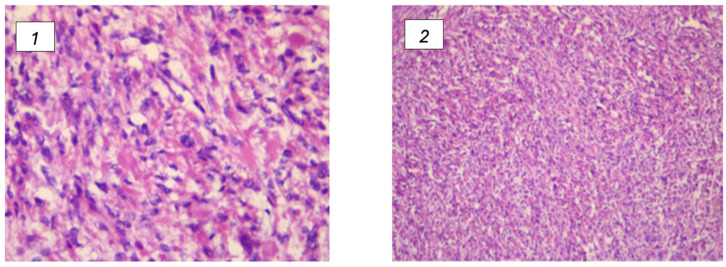
(**1**,**2**) Leiomyomas associated with FH deficiency and their histological changes. (**1**) Presence features with vague nuclear palisading and prominent nuclear pleomorphism; (**2**) the most significant characteristics include marked nuclear pleomorphism and the presence of multinucleated cells. Furthermore, certain nuclei display pseudoinclusions, along with eosinophilic cytoplasmic globules.

FHD can arise from both somatic and germline mutations, each presenting unique clinical and epidemiological features. Uterine leiomyomas are benign smooth muscle tumors of the uterus and are influenced by genetic alterations, including those in the FH gene. Somatic mutations in the FH gene occur within the cells of the tumor itself and are not inherited. These mutations are acquired during the patient’s lifetime and contribute directly to the formation and growth of leiomyomas. Studies have shown that somatic mutations in FH are present in a significant portion of uterine leiomyomas, particularly those that exhibit specific histopathological features such as increased cellularity and atypical nuclei [9] (Figure 2).

Research by Harrison et al. indicates that these mutations are prevalent in a considerable number of FH-deficient uterine leiomyomas, underlining the role of somatic mutations in their development [1]. Other research highlights that somatic mutations account for a small but significant percentage of cases [10,11]: approximately 1–2% of uterine leiomyomas are associated with somatic FH mutations. While this number may seem modest, it is essential to recognize the clinical implications of these mutations, as FH-deficient fibroids tend to exhibit pathological features close to those observed in STUMP and uLMS [5].

Germline mutations, on the other hand, are inherited and present in all cells of the body. FH gene heterozygous mutation predisposes individuals to HLRCC syndrome, which increases the risk for developing multiple leiomyomas as well as renal cell carcinoma. The prevalence of germline FH mutations in the general population is relatively low, but among patients with HLRCC, these mutations are quite common. Studies estimate that germline mutations in FH account for a significant percentage of FH-negative uterine leiomyomas in younger patients (up to 30 years of age) [1,10,12]. Liu C et al. evaluated 153 cases of uterine leiomyomas, detecting a 4.6% (7 of 153 cases) incidence of FH loss of expression in uterine leiomyomas from patients under 30 years of age. Notably, six out of seven FH-negative tumors (86%) were found to harbor FH mutations, with 50% containing a germline mutation [12]. The prevalence of fumarate hydratase deficiency in uterine leiomyomas is influenced by both somatic and germline mutations. Somatic mutations are often identified through histopathological screening and genetic testing of the tumor tissue, whereas germline mutations are typically detected through comprehensive genetic screening of the patient and sometimes their family members. The detection of germline mutations is particularly crucial for early diagnosis and management of HLRCC, which has significant implications for the patient’s overall health due to the associated risk of renal cancer [1,11]. However, the differences between somatic and germline mutations entail different monitoring approaches for the patients, as while somatic mutations primarily contribute to the localized development of leiomyomas, germline mutations indicate a systemic predisposition to tumor development and require vigilant monitoring for associated malignancies, particularly renal cell carcinoma. Germline mutation carriers often undergo regular surveillance and may consider expanded FH screening (for example, young ladies with fibroids) in order to provide an early curative intervention and detection of RCC [13,14].

## 3. Role in Gynecology and Oncology

Patients with FH deficiency are particularly prone to developing benign disease, such as leiomyomas (in the skin and uterus), as well as malignancies, such as renal cell carcinoma (RCC) [14]. Uterine leiomyomas associated with FH deficiency often present in younger women and tend to be multiple and larger compared to sporadic fibroids [4].

Diagnosis of FH-deficient uterine leiomyomas involves a combination of clinical evaluation, imaging, and histopathological examination. Immunohistochemistry (IHC) for FH protein is a crucial diagnostic tool; the absence of FH staining strongly suggests FH deficiency. Genetic testing for FH mutations can confirm the diagnosis and identify at-risk family members [15,16].

Management of FH-deficient leiomyomas includes conventional treatments for fibroids, such as surgical removal or hormonal therapy, but with additional considerations due to the potential for associated malignancies. High-intensity focused ultrasound (HIFU) was found to be a feasible treatment option for patients with FH-deficient uterine leiomyomas. Zhang L. et al. suggests that while HIFU is a promising option for managing FH-deficient uterine leiomyomas, its long-term efficacy may be limited, and patients should be informed about the potential need for reintervention. Indeed, considering the peculiar biological behavior of FH-deficient leiomyomas, surgery remains the recommended treatment for these patients [17]. A case review by Ciccarone et al. reported a diagnosis of uLMS 5 years after HIFU under MRI guidance, showing that HIFU is a safe and non-invasive technique. Despite that, it is not effectively clear if sarcomatous transformation was developed de novo (as several studies suggest [18]) or by left HIFU residual cells that underwent malignant transformation [19]. Patients diagnosed with FH deficiency should undergo regular surveillance for renal tumors and receive genetic counseling to understand the hereditary nature of the condition and its implications for family members [15].

### 3.1. Evidence in Literature

In Table 1, we report selected research and findings related to FH deficiency and its connection to uterine leiomyomas and HLRCC syndrome that we analyzed in this narrative review.

Harrison WJ et al. focused on the identification and characteristics of FH-deficient uterine leiomyomas, particularly in the context of HLRCC syndrome, and found that while most HLRCC patients have FH-deficient leiomyomas, 1% of all uterine leiomyomas also exhibit FH deficiency due to somatic mutations, making it difficult to use FH deficiency as a unique marker for hereditary disease. The study concluded that while FH-deficient leiomyomas are a hallmark of HLRCC, they also occur in a small percentage of unselected leiomyomas due to somatic mutations [1]. Lu E et al. conducted a large-scale study that analyzed FH gene variants in a database of 120,061 individuals, finding FH variants in 1.3% of the individuals tested and a higher-than-expected frequency of autosomal dominant HLRCC variants, occurring in about 1 in every 2668 individuals. This suggests that HLRCC may be more prevalent than previously thought. Individuals with pathogenic autosomal recessive variants did not seem to have an increased risk of cancer, including renal cell carcinoma, when compared to those with negative genetic testing results [11]. In a review article, Kipnis LM et al. underlined the importance of integrating genetic counseling and testing into the management of women diagnosed with atypical uterine leiomyomas, particularly for identifying women with germline FH pathogenic variants who are at increased risk for aggressive renal cell carcinoma [16]. A case study by Bužinskienė D et al. emphasized the critical importance of understanding the metabolic and oncogenic consequences of FH gene mutations. For patients with large leiomyomas and suspected HLRCC, comprehensive genetic counseling, targeted surgical treatment, and vigilant monitoring are essential to manage cancer risks effectively [10]. The study conducted by Jovanović L et al. describes two cases of uterine leiomyomas with rare histomorphological features (marked nuclear atypia, intracellular eosinophilic globules, and abnormal intratumoral vessels) and confirmed that the FH/SDH (succinate dehydrogenase) deficiencies are linked to a familial cancer syndrome such as HLRCC [9]. Understanding the clinical characteristics and typical morphology of leiomyomas in HLRCC compared to those sporadic is crucial in diagnostic flow charts. Uimari O et al. found that women with HLRCC tend to present with more severe and earlier-onset leiomyomas compared to those with sporadic leiomyomas, but without significant differences in fertility outcomes. Women with HLRCC were diagnosed with leiomyomas at a significantly younger age (33.8 years) compared to those with sporadic leiomyomas (45.4 years), and they were frequently more symptomatic at diagnosis (common symptoms include menorrhagia and lower abdominal pain). A higher proportion of women with HLRCC had a greater number of and larger leiomyomas compared to the sporadic group. While HLRCC-related leiomyomas and sporadic leiomyomas share some common histopathological features, HLRCC-related leiomyomas can also exhibit specific features such as nuclear atypia, the presence of eosinophilic nucleoli and globules, absence of hyalinization, higher microvessel density, and distinct Bcl-2 staining pattern, similar to what we observed in leiomyomatosis with bizarre nuclei [5,20]. Fontanges et al. conducted a genomic analysis of array-CGH of 64 patients, of which 37 were leiomyomas with bizarre nuclei (LMBN) and 28 fumarate hydratase deficient leiomyomas (FHDL). The aim of the study was to evaluate the genomic landscape of these subgroups of benign leiomyomas, in relation to challenging differential diagnosis with STUMP and uLMS. The authors divided the cases into three groups: (1) the group of FHD (24/58 cases) with low genomic instability (GI); (2) the group with TP53 deletion (17/58) with high GI; and (3) the group without genomic events (17/58). There were no recurrences or characteristics of uLMS in any of these cases. Results showed that, while a GI < 10 in the LMBN and FHDL is an indicator of benign behavior, 25/51 cases analyzed showed GI > 10 in the absence of clinical recurrence or histopathological malignancy. Therefore, the authors concluded by stating GI < 10 in LMBN remains a benign feature. On the other hand, a GI > 10 alone in LMBN is not sufficient to diagnose malignancy. Notably, in 7 of 24 cases of FHDL, high genomic instability (GI > 10) was observed due to heterozygous loss of RB1, a well-known tumor suppressor gene [5]. Nevertheless, to the best of our knowledge, no study has yet proven a direct correlation between FHD leiomyoma and an increased risk of uLMS.

### 3.2. FH Deficiency and Cancer Risk

FH deficiency alters cellular metabolism acting as oncometabolite. It promotes the Warburg effect, triggering oncogenic pathways. Cancer risk in patients with FH deficiency is significant, due to its key role in HLRCC syndrome and in the lifetime risk of developing renal cell carcinoma (as high as 15–30%) [21,22]. This type of cancer often presents at a younger age and tends to be highly aggressive with early metastatization and poor survival rates in those diagnosed at later stages (III or IV) [7]. Histologically, FHD-related renal cell carcinoma presents papillary growth patterns, cystic tubule formation, eosinophilic cytoplasm, and perinuclear halos [7]. Molecular pathways underlying oncogenesis are better elucidated in the second part of this manuscript. HLRCC patients present multiple skin leiomyomas and, in women, early development of symptomatic uterine fibroids [22]. In the early 2000s, some studies reported an association between FH deficiency leiomyomas and increased risk of developing uterine leiomyosarcoma [22]. However, Harrison et al. suggested that FH deficiency in leiomyomas might have been over-interpreted because of their morphological features like uterine leiomyosarcomas (uLMS) (hypercellularity and tendency for symplastic nuclear atypia), leading pathologists to misdiagnose benign leiomyomas as malignant leiomyosarcomas [1]. More recently, Chapel et al. suggested that FH deficiency in the context of uLMS might not to be a primary event leading to malignancy. Instead, FH inactivation might be a consequence of genomic instability, such as large chromosomal deletions or loss of heterozygosity (LOH), characteristic of uLMS [23]. This evidence led to the belief that FH deficiency could be involved in more complex genetic changes in the tumor, rather than be a key factor in driving uLMS. Nevertheless, while FHD has been identified as a distinct feature in a subgroup of uterine leiomyoma, its role in uLMS remains unclear, and further research is needed to better understand its potential landscape features. So far, no study has proven the linkage between FH loss of expression and leiomyosarcoma transformation. 

### 3.3. Management and Surveillance

Management of FDH deficiency-related conditions requires a multidisciplinary approach, including metabolic interventions to manage symptoms and targeted therapies aimed at inhibiting HIF pathways. Surgical interventions may also be necessary to remove tumors. Understanding the molecular and genetic basis of FH deficiency is essential for the development of effective diagnostic and therapeutic approaches. Continued research into the mechanisms underlying FH deficiency and its role in oncogenesis is necessary to improve outcomes for patients with this condition [7,10]. Given the significant cancer risk, prevention and early detection strategies are critical for managing patients with FH deficiency. Genetic testing is essential for diagnosing FH mutations, especially in individuals with a family history of HLRCC or those presenting with multiple cutaneous and uterine leiomyomas. Once a diagnosis is confirmed, regular surveillance for renal cell carcinoma is recommended. This typically involves annual abdominal imaging, such as MRI or CT scans, starting at an early age. The goal is to detect renal tumors at an early, more treatable stage. Preventive measures also include counseling patients and their families about the hereditary nature of the condition. Patients are advised of the importance of regular monitoring and the potential need for prophylactic measures, such as surgical intervention for symptomatic leiomyomas or early-stage renal tumors [24,25,26].

The biological significance of FH deficiency in uterine leiomyosarcoma (uLMS), including its relationship with germline FH mutations and its impact on patient outcomes, is still under study. A Cohort Study conducted by Chapel DB et al. analyzed 348 cases of uLMS, and 2% (7 cases) were identified as FH-deficient. The study showed that FH deficiency does not exclude the possibility of malignancy in uterine smooth muscle tumors; however, given the rarity of these tumors, the study calls for a larger, multi-institutional effort to better characterize FHD uLMS at both clinicopathologic and molecular levels [23]. 

## 4. Future Perspectives

Future research should focus on several key areas to improve the understanding and management of FH deficiency in gynecologic oncology. New perspectives are summarized in Table 2.

Biomarker Development: Identifying reliable biomarkers for early detection of FH-deficient tumors can enhance screening programs and allow for timely interventions. Proteomic and metabolomic profiling may yield potential biomarkers that reflect the metabolic alterations associated with FH deficiency. Use of liquid biopsy could be considered in the field to obtain a feasible and quicker tool for the identification of genetic FH loss, eventually identifying circulating biomarkers which could be useful for target therapies.

Targeted Therapies: Developing targeted therapies that specifically address the metabolic and epigenetic consequences of FH deficiency is promising. Inhibitors of HIFs or agents that can reduce fumarate levels may prove effective in slowing down or reversing tumor progression in affected individuals. Sulkoswski et al. have demonstrated that accumulation of fumarate and succinate as a result of FH deficiency can impair homologous recombination and DNA repair, like what we observed in BRCA1/BRCA2 mutated breast cancer and ovarian cancer [27]. This suggests the potential therapeutic role of Poly ADP Ribose Polymerase (PARP) inhibitors or medications which target the downstream HIF-1α and HIF-2α pathway activation in the management of FHD-related malignancies. The PD-1 blockade has improved treatment for advanced clear cell renal cell carcinoma (ccRCC), but the factors influencing the response to therapy are not fully understood [28]. Braun, D.A et al. found that traditional markers, such as tumor mutation burden and CD8+ T cell infiltration, did not predict treatment response. However, they identified chromosomal alterations linked to either response or resistance to the PD-1 blockade [27]. Tumors with high CD8+ T cell infiltration were generally depleted of beneficial PBRM1 mutations and showed an increased loss of chromosomal region 9p21.3, which was associated with resistance [28]. These findings highlight how the combination of immune features and genetic mutations can affect the success of the PD-1 blockade in ccRCC [28]. On the other hand, it is important to remember that the PD-1 blockade can also determine autoimmune toxicity, such as dermatologic (rash, pruritis, and vitiligo), endocrine (thyroiditis, hypophysitis), hepatitis, colitis, and others rare toxicities (neurological, ocular, and cardiac). Most of these immunotherapy-related toxicities are managed by corticosteroid treatment [29].

Clinical Trials: Conducting clinical trials to test the efficacy of novel therapeutic agents and treatment regimens tailored for FH-deficient tumors is crucial. These trials should explore the benefits of combining targeted therapies with existing treatment modalities, such as surgery, radiation, and chemotherapy. Sintilimab, a PD-1 inhibitor, is actually studied in the NCT04146831 Phase II trial (unknown status on clinicaltrials.gov) in second-line treatment in FH-deficient renal cell carcinoma settings. The NCT01130519 Phase II trial (active, not recruiting) aims to study the effectiveness of the combination of Bevacizumab and Erlotinib in subjects with advanced hereditary leiomyomatosis and renal cell cancer (HLRCC) or sporadic papillary renal cell cancer. No results are available to our knowledge.

Genetic Counseling and Screening Programs: Expanding genetic counseling services and implementing comprehensive screening programs for individuals with a family history of HLRCC can help in early diagnosis and management. This approach can also provide valuable data on the prevalence and clinical outcomes of FH deficiency in various populations.

## 5. Conclusions

Fumarate hydratase deficiency is an emerging area of interest in gynecologic oncology due to its association with hereditary leiomyomatosis and renal cell carcinoma (HLRCC) syndrome, which includes a predisposition to developing uterine fibroids, renal cell carcinoma, and occasionally, aggressive uterine cancers like leiomyosarcoma. The deficiency of FH, a critical enzyme in the tricarboxylic acid cycle, leads to the accumulation of fumarate, which subsequently promotes oncogenic pathways and epigenetic alterations, contributing to tumorigenesis. Patients with FH deficiency are at increased risk of aggressive forms of malignancies. Thus, early detection and genetic screening for FH mutations can aid in identifying individuals at risk and implementing appropriate surveillance and management strategies. The recognition of FH deficiency’s role in gynecologic oncology underscores the need for personalized treatment approaches and targeted therapies which can exploit this vulnerability. In summary, addressing the challenges posed by FH deficiency in gynecologic oncology requires a multifaceted approach encompassing early detection, targeted therapies, clinical trials, and ongoing research aimed at also identifying the potential role of FH deficiency in leiomyoma transformation in leiomyosarcoma. Advancements in these areas hold the potential to significantly improve outcomes for patients with FH-deficient gynecologic cancers. 

## Figures and Tables

**Table 1:** List of some relevant published articles understanding the relationship between FH deficiency and uterine leiomyomas, particularly in the context of HLRCC syndrome.

Year	Main Author	Type of Article	N° of Patients	Type of Tumor	Main Findings
2016	Harrison WJ	Research article	Not specified	Uterine Leiomyomas	FH-deficient leiomyoma linked to HLRCC syndrome [1]
2021	Uimari O	Research article	97	Uterine Leiomyomas	Clinical characteristics and typical morphology of leiomyomas in HLRCC compared to those sporadic [20]
2022	Lu E	Research article	120,061	Uterine Leiomyomas, renal cell carcinoma	Germline FH variants associated with HLRCC syndrome [11]
2024	Kipnis LM	Review article	Not specified	Uterine Leiomyomas, renal cell carcinoma	Expert-reviewed summary on HLRCC syndrome [16]
2024	Bužinskienė D	Case report	1	Uterine Leiomyomas, renal cell carcinoma	Details familiar cancer syndromes caused by FH and SDH mutations [10]
2024	Jovanović L	Research article	2	Uterine Leiomyomas	Identified specific histological features of FH deficiency in uterine leiomyomas [9]
2024	Fontanges Q	Research article	64	Uterine Leiomyomas	Genomic landscape of benign leiomyomas in relation to differential diagnosis with STUMP and uLMS [5]

**Table 2:** New perspectives for better management of FH deficiency.

Perspectives	Objectives
Biomarker Development	- Identification of reliable biomarkers for early detection of FH-deficient tumors; - Proteomic and metabolomic profiling to detect metabolic alterations (e.g., fumarate accumulation); - Liquid biopsy as a quick, non-invasive method to detect genetic loss of FH.
Target Therapy	- HIF inhibitors and agents that reduce fumarate levels could slow down tumor progression; - PARP inhibitors may restore DNA repair in FH-deficient tumors, similar to BRCA-related cancers; - PD-1 blockade for ccRCC treatment.
Clinical Trials	- Sintilimab (PD-1 inhibitor) is in Phase II (NCT04146831) for FH-deficient ccRCC; - Study on Bevacizumab + Erlotinib in HLRCC (NCT01130519)—currently not recruiting.
Genetic Counseling and Screening Programs	- Expanding genetic counseling and screening programs for individuals with a family history of HLRCC; - Screening programs aid in early diagnosis and better management of FH-deficient patients.

## Abbreviations

The following abbreviations are used in this manuscript:

FH	Fumarate hydratase
HLRCC	Hereditary leiomyomatosis and renal cell carcinoma
STUMP	Smooth muscle tumor of uncertain malignant potential
uLMS	Uterine leiomyosarcoma
FHD	Fumarate hydratase deficiency
HIFs	Hypoxia-inducible factors
NADH	Nicotinamide adenine dinucleotide
(FAD)H2	Flavin adenine dinucleotide
ATP	Adenosine triphosphate
IHC	Immunohistochemistry
HIFU	High-intensity focused ultrasound
SDH	Succinate dehydrogenase
LBMS	Leiomyomas with bizarre nuclei
FHDL	Fumarate hydratase deficient leiomyomas
GI	Genomic instability
LOH	Loss of heterozygosity
PARP	Poly ADP Ribose Polymerase
ccRCC	Clear cell renal cell carcinoma
